# An inventory of collaborative medication reviews for older adults - evolution of practices

**DOI:** 10.1186/s12877-019-1317-6

**Published:** 2019-11-21

**Authors:** A. Kiiski, M. Airaksinen, A. Mäntylä, S. Desselle, A. Kumpusalo-Vauhkonen, T. Järvensivu, M. Pohjanoksa-Mäntylä

**Affiliations:** 1 Clinical Pharmacy Group, Division of Pharmacology and Pharmacotherapy, Faculty of Pharmacy, University of Helsinki , PO Box 56, 00014 Helsinki, Finland; 2 Finnish Medicines Agency Fimea, PO Box 55, 00034 Helsinki, Finland; 3 Present address: Kärsämäki Pharmacy, Frosteruksenkatu 4, 86710 Kärsämäki, Finland; 4 College of Pharmacy, Touro University California, 1310 Club Drive Mare Island Vallejo, California, CA 94592 USA; 5 Present address: Vieremä Pharmacy, Petterintie 2, 74200 Vieremä, Finland; 6 Aalto University, PO Box 11000, FI-00076 AALTO Espoo, Finland

**Keywords:** Collaborative medication review, Comprehensive medication review, Concordance and compliance review, Adherence review, Prescription review, Medicines optimization, Older adults

## Abstract

**Background:**

Collaborative medication review (CMR) practices for older adults are evolving in many countries. Development has been under way in Finland for over a decade, but no inventory of evolved practices has been conducted. The aim of this study was to identify and describe CMR practices in Finland after 10 years of developement.

**Methods:**

An inventory of CMR practices was conducted using a snowballing approach and an open call in the Finnish Medicines Agency’s website in 2015. Data were quantitatively analysed using descriptive statistics and qualitatively by inductive thematic content analysis. Clyne et al’s medication review typology was applied for evaluating comprehensiveness of the practices.

**Results:**

In total, 43 practices were identified, of which 22 (51%) were designed for older adults in primary care. The majority (*n* = 30, 70%) of the practices were clinical CMRs, with 18 (42%) of them being in routine use. A checklist with criteria was used in 19 (44%) of the practices to identify patients with polypharmacy (*n* = 6), falls (*n* = 5), and renal dysfunction (*n* = 5) as the most common criteria for CMR. Patients were involved in 32 (74%) of the practices, mostly as a source of information via interview (*n* = 27, 63%). A medication care plan was discussed with the patient in 17 practices (40%), and it was established systematically as usual care to all or selected patient groups in 11 (26%) of the practices. All or selected patients’ medication lists were reconciled in 15 practices (35%). Nearly half of the practices (*n* = 19, 44%) lacked explicit methods for following up effects of medication changes. When reported, the effects were followed up as a routine control (*n* = 9, 21%) or in a follow-up appointment (*n* = 6, 14%).

**Conclusions:**

Different MRs in varying settings were available and in routine use, the majority being comprehensive CMRs designed for primary outpatient care and for older adults. Even though practices might benefit from national standardization, flexibility in their customization according to context, medical and patient needs, and available resources is important.

## Background

Polypharmacy and associated high medication costs commonly occur within a small proportion of medicine users, a majority of which are≥65 years old [1]. As polypharmacy is associated with increased risk of medication-related problems, different medicines optimization strategies are required for various patients [1–3]. According to the National Institute of Health and Care Excellence (NICE) in the United Kingdom (UK), medicines optimization is a person-centered approach to safe and effective medicines use to ensure that people obtain the best possible outcomes from their medicines [2].

Collaborative medication reviews (CMRs) are one of the methods in medicines optimization to prevent inappropriate medication use and to increase adherence [2, 4–10]. CMR is an internationally recognized term referring to medication review practices involving pharmacists as reviewers of the medication in close collaboration with other health care professionals. CMR practices differ within countries and internationally, for example by the context and the patient groups they are designed for, which healthcare professionals are involved, the degree of collaboration, and the degree of patient involvement in the process [5–7, 9, 11–22]. Currently, CMR practices are under development in many countries, with the most evidence of impact on patient and service outcomes, for example, the appropriateness of the medication and readmission rates, respectively, coming from pioneering countries, such as the USA, Australia, and UK [5–7, 9, 11–21].

Finland has one of the most rapidly aging populations in the world. As a consequence, resent Government Programs have focused on finding strategies for managing risks and costs in geriatric pharmacotherapy in outpatient and inpatient care [23, 24]. CMRs have been prioritized in the Government Program 2015–2019 for implementing rational pharmacotherapy as a part of substantial healthcare reform [25, 26]. Development of collaborative medication review practices was initiated in Finland in 2005 as part of a national program aimed to strengthen community pharmacists’ involvement in patient care [19, 27]. For that purpose, long-term accreditation training (1.5 years alongside work) for practicing pharmacists was initiated to attain collaborative comprehensive medication review competency [27]. Since then, CMR practices involving pharmacists and other health care professionals have evolved in outpatient and inpatient care [28–31]. Thus, Finland provides an example of a country with no previous history of patient-oriented clinical pharmacy practice where initiation of CMRs and related accreditation training have remarkably fostered remodeling the pharmacists role in patient care. Sharing experiences could be helpful in other countries seeking to evolve pharmacists’ roles in a similar manner. Furthermore, Finland has benchmarked advanced CMR practices in other countries, particularly in Australia and USA in the early phase of starting the CMR accreditation training in 2005 [19, 27]. Despite growing evidence of the positive outcomes (the appropriateness of the medication and readmission rates, respectively) associated with CMRs [5–7, 9, 11–22, 32], there has not been a comprehensive evaluation of their implementation on a nationwide basis. Detailed descriptions of CMR procedures performed are important, as the intensity of their conduct and range of services provided (for example, prescription reviews vs. clinical medication reviews) might be associated with varying productivity, or degree of patient outcomes achieved. The aim of this study was to identify and describe the state of CMR practices in Finland after 10 years of development.

## Methods

### Study design and setting

In seeking to create an inventory to describe the state of CMR practices in Finland, the researchers conducted a web-based open call by the Finnish Medicines Agency (later Fimea) in April–May 2015. The call was targeted to all health care professionals involved in collaborative teams reviewing medications of older adults in any health care context. The inventory was part of Fimea’s long-term program to promote rational medicine use of older adults, and the Ministry of Social Affairs and Health funded a research project on medicines optimization for older adults (ILMA) [10].

### Inventory instrument

The inventory instrument (Additional file 1) was developed in four phases: 1) drafting the first version of the instrument; 2) modifying the content of the instrument with help of the medication review experts; 3) modifying the instrument to cover all health care contexts; and 4) pilot testing the instrument.

#### Phase 1

The first draft of the inventory instrument was adapted from questions previously used 1) by Fimea to investigate challenges and solutions in medication management of older adults [13], 2) by our research group in an inventory of medication review practices in European Union (EU) countries [33], and 3) in a systematic review of CMR practices and their effectiveness in outpatient care [10]. The questions covering typical phases of a medication review process were the following (See also Additional file 1): 1) Where, how and by whom is a patient with medication-related problems identified? 2) Which health care professionals are involved in reviewing the medication? 3) How is the patient involved? 4) How do different health care professionals and organizations communicate patient information in different phases of the medication review process? 5) Where are the patient’s medications reviewed (the site/venue and phase of the patient’s care pathway)? 6) How is the actual review of the medications performed? 7) How is the follow-up of implementing medication changes organized? 8) Is the practice based on any previous medication review procedure and/or theory? 9) What kind of tools are used in reviewing medications (electronic and/or manual)? and 10) What is the context of the practice? Additionally, the inventory solicited data on the medication-related issues reviewed, involvement of proxies in the medication review, and treatment follow-up practices in usual patient care.

Finally, the respondents were asked to indicate the comprehensiveness of their practice according to Clyne et al.(2008) [34], which is a National Health Service (NHS)-based categorization widely used in the UK and elsewhere as criteria for standardizing CMRs [13, 22]. It categorizes CMR practices into the following three categories: 1) prescription reviews; 2) concordance and compliance reviews; and 3) clinical medication reviews. The categorization takes into consideration objectives of the review (addressing technical issues, the patient’s medicines taking behavior, and the patient’s use of the medicines in the context of his/her clinical condition), the used patient information sources, medications reviewed (only prescription medicines, both prescription and non-prescription medicines and other complementary products), and patient involvement in the review (not involved, usually involved or always involved). Respondents were asked to identify the comprehensiveness of their CMR practice by defining its purpose, i.e., whether it was for: 1) technical review of prescriptions / list of medications (i.e., prescription review); 2) review of the prescriptions, the patient is involved to discuss medication taking and support adherence (i.e., adherence/concordance review); or 3) review of the medications in the context of the patient’s clinical condition (i.e., clinical medication review).

#### Phase 2

Six medication review experts from different health care contexts (hospital, community pharmacy and assisted living) and the Association of Finnish Pharmacies (AFP) were consulted when forming the final inventory instrument. Questions related to patients and health care professionals’ involvement in the medication review process and in medication-related issues reviewed were modified according to their feedback. New questions were added relating to following up implementation of medication changes and involving proxies in medication reviews.

#### Phase 3

Questions were rephrased according to experts recommendations to enable to reflect on CMR practices in different contexts. Some open-ended questions were also changed to structured questions to reduce response burden.

#### Phase 4

The inventory instrument was piloted for face and content validity and technical functionality by the same experts involved in developing it. Changes were minor at this phase, limited to wording and grammar, not content. The final inventory instrument is presented in Additional file 1.

### Data collection

The inventory was conducted using a snowballing approach and an open call via the Finnish Medicines Agency (Fimea’s) website where the invitation letter with a link to the inventory instrument was openly accessible in April–May 2015. The link was also widely disseminated via expert networks and mailing lists. Receivers of the invitation were encouraged to report as many CMR practices as they were involved in and to forward the link to their networks [35]. This data collection method was chosen, as no previous data or register was available about the CMR practices and healthcare providers involved in Finland. Two reminders were sent to the same receivers than the original link 2 weeks after opening the call (beginning and end of the week). Responding was voluntary, and respondents did not receive any incentives. Responding to the survey was considered as giving informed consent.

### Data analysis

Data gathered by structured questions were quantitatively analyzed for descriptive statistics using frequencies and percentages by Statistical Package for the Social Sciences (SPSS-21) and Excel-program (2013). Open-ended questions (indicated in the Additional file 1) were analyzed qualitatively by inductive thematic content analysis, meaning that all themes arise directly from the survey responses [36]. The narrative responses were read, encoded and categorized into conceptual themes. The frequencies of appearance of each categorized theme in the responses were counted, and the results were presented as frequencies and percentages to obtain understanding of the medication review process phases most commonly performed. Incomplete questions were analyzed as such (information not available).

For the analysis, the reported CMR practices were categorized into three categories according to their comprehensiveness by using Clyne et al’s typology [34]. First, the respondents were asked to categorize their CMR practice according to Clyne et al’s typology by selecting an option from the structured list that best fitted the purpose of their practice (Additional file 1). To validate the categorization, the researchers independently performed another categorization by using all the provided information on the practice, with a special emphasis on patient information sources, medications, patient involvement and objectives of the review. Active patient involvement was required in type 3 practices (clinical medication review). In Clyne et al., the minimum criteria for active patient involvement is that the patient is present [34]. Our interpretation of this criteria is that at minimum the patient should be interviewed. Finally, the categorization performed by the researcher was compared with that performed by the respondent. In cases of discrepancies, the categorization by the researcher was used.

## Results

### Medication review practices

**Fig. 1:**
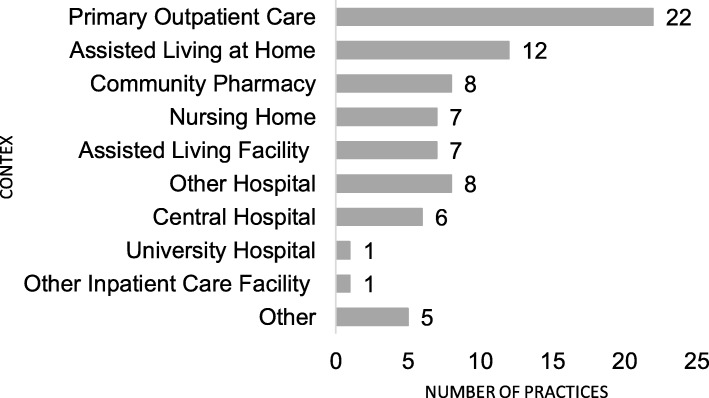
Health care context of the reported collaborative medication review practices for older adults in 2015 (*n* = 43, 15 of the practices were conducted in multiple contexts)

In total, 43 CMR practices were identified by 38 respondents, of which 22 (51%) practices were designed for older people in primary care (Fig. 1). Almost all practices (*n* = 42/43) were established after the year 2005. The majority (*n* = 30, 70%) of the practices were comprehensive clinical medication reviews, involving patients in the review process and concentrating on clinical evaluation rather than reviewing medication lists. Seven (16%) of the practices were concordance and compliance reviews, and two (5%) were prescription reviews. It was not possible to categorize four (9%) of the practices according to Clyne el al’s typology [34], because they were comprised of only one phase of the review process or described use of a tool in reviewing medications (for example, a tool to identify patients with risk factors for medication-related problems). In 40 out of 43 practices (93%) the respondent and the researcher agreed on the categorization of the practice: 3 practices (7%) were graded by the respondents to be more comprehensive than did the researchers. The majority of the practices (*n* = 29, 67%) were targeted to outpatients, 8 (19%) to inpatients and 6 (14%) to both types of patients. Fifteen of the practices (35%) were used in at least two different health care contexts (Fig. 1). Of the practices, 28 (65%) were government or municipality funded. Most of the practices (*n* = 31, 72%) were established between 2013 and 2015. Eighteen of them (42%), were in routine use, 18 (42%) were in a pilot phase, 4 (9%) under development, and one (2%) was in a planning phase.

### Identification of patients with medication-related problems

A checklist with criteria was used in 19 (44%) of the practices to identify patients with medication-related problems. Twelve of them included explicit criteria that varied considerably between practices (Table 1). The commonly used criteria were 1) polypharmacy expressed as number of medicines in use (*n* = 6), 2) number of falls (*n* = 5), and 3) renal dysfunction (n = 5). Information about the identified patient with medication-related problems was transmitted via electronic health records (EHRs) (*n* = 11, 26%), interpersonal communication (*n* = 7, 16%) or direct contact with the physician via different communication means, (n = 6, 14%). Most commonly, patients with medication-related problems were identified by nurses (*n* = 39, 91%), pharmacists (*n* = 32, 74%) and physicians (*n* = 28, 65%).

**Table 1:** Identification criteria for patients with medication-related problems (includes 12 practices that were reported to have explicit identification criteria). One checklist consists of multiple criteria

Identification criteria	n	% of practices having criteria (*n* = 12)	% of all practices (*n* = 43)
Polypharmacy	6	50	14
8 medicines in use	1	8	2
7 medicines or more	3	25	7
Not defined in detail	3	25	7
Fal^a^	5	42	12
Renal dysfunction	5	42	12
Low GFR^b^	2	17	5
GFR under 60	1	8	2
GFR under 50 (60)	1	8	2
Not defined in detail	1	8	2
Dizziness	4	33	9
Age	4	33	9
> 75 years	3	25	7
Older person, but age not explicitly defined	1	8	2
Poor response to treatment	4	33	9
Poor adherence	3	25	7
Acute decline in general condition	3	25	7
Increased need to use health services	2	17	5
Ortostatism	1	8	2
Delirium or disturbance of consciousness	1	8	2
Constipation	1	8	2
Dry mouth	1	8	2
Concerns regarding possible adverse drug reactions	1	8	2
Unclear or long medication list	1	8	2
Multiple psychotropic medicines in use	1	8	2
Multiple interactions	1	8	2
Liver dysfunction	1	8	2
Living at home	1	8	2

^a^Respondents did not specify the timeframe of the fall

^b^*GFR* glomerulus filtration rate

### Conducting collaborative medication reviews

CMRs were most commonly conducted at the point of prescribing (*n* = 10, 23%), on the ward (*n* = 10, 23%), or in assisted living (*n* = 5, 12%). Nurses (*n* = 40, 93%) and physicians (*n* = 39, 91%) were involved in most of the practices. Other health care professionals involved were pharmacists (*n* = 35, 81%), practical nurses (a title in Finland for nurses having a 3-year vocational education that focuses on supportive and technical nursing) (*n* = 23, 53%), and physiotherapists (*n* = 6, 14%). In half of the practices, pharmacist reviewed medications (*n* = 22, 51%) (Table 2). Nurses had central contribution throughout the reviewing process. Most of the responsibilities was shared and conducted collaboratively. Phases that were acted alone by particular professionals were most commonly decisions on medication changes by physicians (*n* = 17, 40%), follow-up of the medication changes by practical nurses (*n* = 8, 19%), and medication reviews by pharmacists (*n* = 20, 47%).

**Table 2:** Healthcare professionals involved in the collaborative medication review process (*n* = 43 practices) and process phases acted alone by particular professionals (*n* = 43 practices)

Healthcare professional’s contribution	Practitioner acting alone in the phase of the process		Total number of practices (*N*=43)	
PHYSICIAN	n	%	n	%
Patient identification	1	2	28	65
Decision making on medication changes	17	40	17	40
Follow-up of medication changes	1	2	8	19
Patient counseling on medication changes	2	5	8	19
Medication reconciliation	-		2	5
Instructing other health care professionals on how to follow-up	1	2	1	2
Patient examination	1	2	1	2
Medication review	1	2	1	2
Charting admission notes for further review	1	2	1	2
Developing the MR practice	-		1	2
PRACTICAL NURSE				
Patient identification	3	7	39	91
Patient counseling on medication changes	5	12	15	35
Follow-up of medication changes	8	19	15	35
Informing other care team members about the patient’s health condition	6	14	8	19
Implementation of medication changes	2	5	8	19
Medication review	-		3	7
Patient interview	1	2	2	5
Medication reconciliation	2	5	2	5
Identification of medication-related problems	1	2	1	2
Ordering additional patient testing	-		1	2
Developing the MR practice	-		1	2
NURSE				
Patient identification	6	14	17	40
Follow-up of medication changes	1	2	5	12
Patient interview	-		1	2
Informing other health care professionals about the patient’s health condition	-		1	2
Medication review	1	2	1	2
Ordering additional patient testing	-		1	2
Medication reconciliation	-		1	2
PHARMACIST				
Patient identification	3	7	32	74
Medication review	20	47	22	51
Follow-up of medication changes	2	5	6	14
Patient interview	5	12	5	12
Developing the practice	-		1	2
Patient counseling on medication changes	-		1	2
PHYSIOTHERAPIST				
Evaluation of the patients ability to function	1	2	1	2
Patient identification	-		1	2
Information not available			11	26

**Fig. 2:**
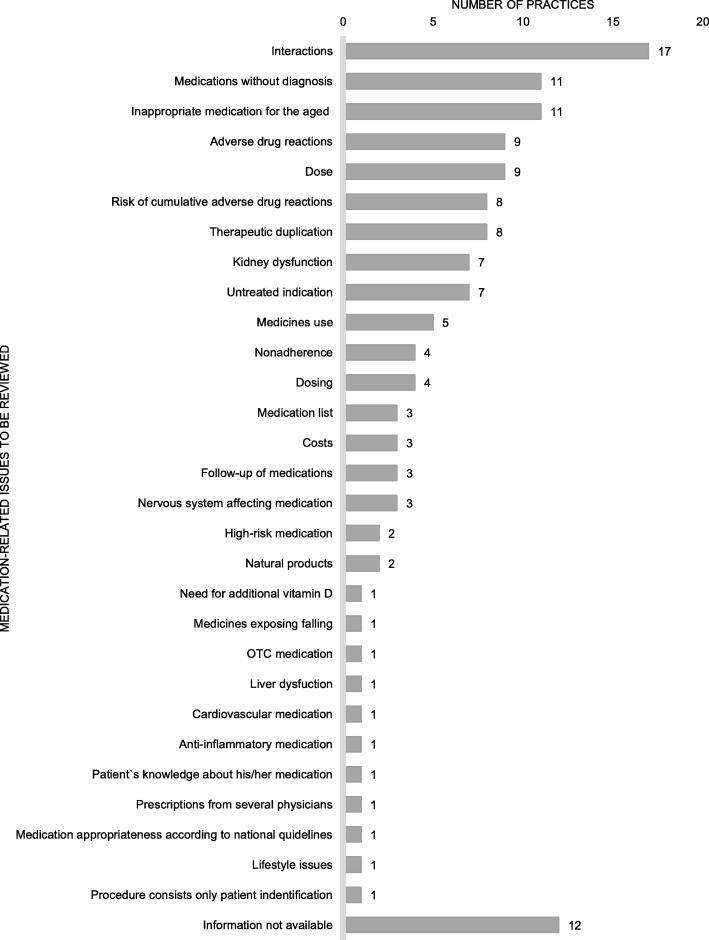
Medication-related issues to be reviewed in the practices (practices *n* = 43, several aspects can be reviewed in the same medication review)

**Fig. 3:**
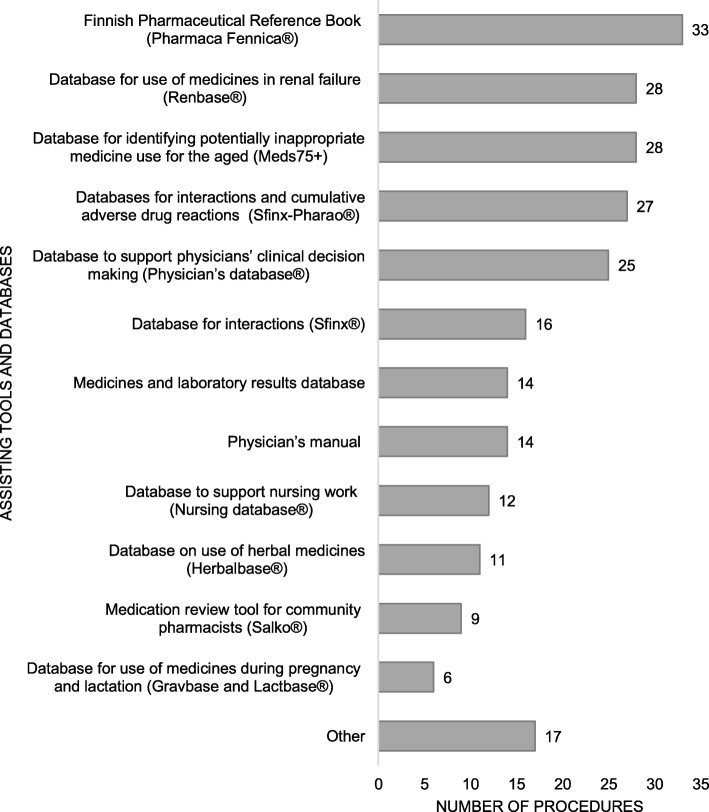
Assisting tools and databases in medication review practices (*n* = 43), more than one tool and/or database can be used in the same practice

CMRs most commonly focused on interactions, medications without diagnosis, and potentially inappropriate medications for older adults (PIM’s) (Fig. 2). Few practices considered appropriateness of the medication according to national current care guidelines, self-medication and use of natural products, or patient adherence to the medications. Electronic National Reference Book and databases for medicine use in renal dysfunction, for identifying potentially inappropriate medicine use for the aged, interactions and adverse drug reactions, and the clinical decision-support system for physicians were frequently used to assist in reviewing medications (Fig. 3). Generally, more than one tool or database was utilized (*n* = 35, 81%), and the most common combination was Electronic National Reference Book and database for medicine use in renal failure (*n* = 28, 65%).

Most commonly, the physician decided on medication changes after hearing other health care professionals (*n* = 24, 56%), or decisions were made in a team meeting (*n* = 19, 44%). Decisions were seldom made together with the nurse and the physician (*n* = 1, 2%), in the ward (*n* = 1, 2%), or via remote conferencing (*n* = 1, 2%).

Information was transferred between healthcare professionals or organizations most commonly via patient information systems (*n* = 19, 44%), as a written report (*n* = 9, 21%), by telephone (*n* = 7, 16%), through conversation (*n* = 7, 16%) or electronically (*n* = 7, 16%). Patients received information most commonly as a written summary (*n* = 16, 37%) or via conversation (*n* = 15, 35%) with one or more health care professionals.

### Patient involvement

Patients were actively involved in 32 (74%) of the CMR practices inventoried, mostly as a source of information via patient interview (*n* = 27, 63%), but seldom in the implementation of medication changes. A medication care plan was discussed together with the patient in 17 practices (40%), and in eight practices (19%) patients updated the medication list by themselves. A home visit was included in 15 practices (35%). Proxies were involved in the process most commonly as a source of patient information (*n* = 18, 42%), or information was relayed to them about the patient’s care (*n* = 7, 16%).

### Follow-up

Medication care plans were established routinely as usual care systematically to all or selected patient groups in 11 of the practices (26%) or to some patients in 11 of the practices (26%). The medication list was reconciled as usual and customary care systematically to all or selected patient groups in 15 of the practices (35%) or to some patients in four of the practices (9%) routinely. In nearly half of the practices (*n* = 19, 44%), information was missing concerning the ways in which the effects of the medication changes were followed up. When reported, (*n* = 24, 56%), effects were most commonly followed up in a routine control (*n* = 9, 21%) or in a separate follow-up appointment (*n* = 6, 14%). Information about follow-ups was registered into patient information systems (*n* = 6, 14%) or into a care plan (*n* = 3, 7%).

## Discussion

This study was the first inventory of CMR practices in Finland. The inventory identified a remarkable number of practices developed within a decade, of which the majority were comprehensive clinical medication reviews. Nearly half of the practices were in routine use in various healthcare contexts in 2015, which may indicate that CMRs are becoming a part of usual and customary care. This inventory provides valuable information for further optimization of the existing practices and development of the new ones that were numerous under way in 2015 when the inventory was conducted. The inventory describes practices and the manner in which they take place in actual patient care and in more detail than previous literature [4–9, 37, 38]. Based on the study findings, CMR practice issues requiring more attention and standardization are: 1) criteria used for identifying older adults needing medication review, 2) enhancing patient involvement in implementing the therapeutic plan, particularly when medication changes are implemented, and 3) enhancing follow-up of medication changes.

Potentially greater proportions of comprehensive CMR practices reported in Finland compared with other countries may be due to their origin in such practices as Home Medicines Reviews (HMRs) in Australia [10, 13, 19, 27]. Benchmarking and cooperating with other countries having more advanced CMR practices were instrumental for getting started by learning from their experiences. For example, the structure of Finland’s CMR process was inspired from Australia’s HMR program [27]. The HMR process is initiated by the physician and conducted as a teamwork. In a home visit the pharmacist reviews the patient’s medications. The findings are discussed during a collaborative case conference. The patient is central in the development and implementation of the medication management plan aiming to maximize the patient’s benefits from the medications and prevent medication-related problems.

The development of the first Finnish comprehensive medication review procedures was initiated in 2005 as part of a long-term continuing education (1.5 years alongside work) providing accredited medication review competence for practicing pharmacists [19, 27]. The education revolves around patient-oriented pharmacotherapy with special focus on geriatric care, medication review principles and interprofessional collaboration. In many other countries, the development of practices has begun from medication list reviews (i.e. prescription reviews), which have been extended to more comprehensive procedures [13]. Given that a wide variety of CMR practices was identified, national standardization might be considered to ensure quality in patient care. However, it should be recognized that different practices are needed in different contexts with varying resources and patients needs [30, 39].

Compared with CMR practices in other countries, nurses, particularly practical nurses, seem to have a stronger role in Finland [4–10]. Practical nurses’ contribution was especially strong in identifying older people needing medication reviews, but they also contributed commonly by counseling patients on medication changes and following up their implementation. This may be due to the fact that practical nurses are the health care providers working most closely with older patients in primary care in Finland, for example in home care and assisted living. If they notice any problems with their clients’ medications, they are expected to report their findings to the nurse, and when needed, involve the physician in solving the problems [40]. Our findings indicate that also pharmacists were quite often involved in identifying patients with medication-related problems and reviewing their medications. This has become more common since medication review accreditation training was started for pharmacists [41]. Still, the local medication management processes need to be better coordinated to make better use of the existing resources [30, 31, 33].

The criteria for identifying older patients with medication-related problems were missing from the majority (72%) of the reported practices. Only 12 practices included the criteria which were commonly 1) the number of medicines in use, 2) falls and 3) renal dysfunction. In addition to these, a long and diffuse list of individual criteria, each of them appearing in one of the procedures was found. This suggests that consensus should be reached on issues contributing to clinically significant medication-related problems in older adults. Evidence on appropriate criteria is still scarce, but it appears that for example the number of medicines in use is not a priority criterion although widely used [40]. A recent pilot study suggests that priority criteria should include symptoms that may be drug-induced such as drowsiness, skin rash or itch, dizziness and urination problems; having more than one physician involved in patient care; and more than one fall in the past 12 months [40]. Further research is needed on the identification criteria to find the older patients benefitting the most from CMRs.

In this inventory, patients were reported to be involved in the implementation of the medication changes, not only serving as a source of background information, in several practices at different types of the CMRs. It is essential to continue emphasizing and facilitating patient involvement throughout the medication review process because involving patients in healthcare decision-making has found to lead to enhanced adherence and potentially better treatment outcomes [42]. This empowering trend has been highlighted in the medicines policy in Finland and elsewhere in the EU [43, 44].

The reports provided little information about follow-ups on medication changes. Medication review may have little effect on patient care if the follow-up is missing or is inadequate, or there is no agreement about the implementation of the medication changes to patient care. Follow-up of medication changes is an area for improvement in Finland, but likely for other countries, as well [10].

### Limitations

While the inventory was derived from practices in Finland, lessons learned can be worthy of consideration by practitioners and policymakers in other countries. Due to the snowballing approach used, this inventory does not necessarily cover all CMR practices in Finland at the time of the inventory. However, there were rich data received via this method from various CMR practices in different healthcare settings.

The data reflect evolution of CMR practices by 2015. Since then, the number and variety of practices have evolved, possibly in a growing pace because CMRs were recommended by the Government Program in 2015 for mitigating health challenges posed by the rapidly aging population. Further research is recommended to follow up the most recent developments within the last 4 years. Future research is also recommended to model interactions within the CMR practices and define outcome variables and their predictors.

The content validity of the inventory instrument was carefully examined; however, true construct validity was not established. Although open-ended questions enabled the informants to describe practices in their own words, this might have increased response burden to some informants, thereby influencing accuracy and completeness of their responses.

### Practical implications

This first inventory conducted in 2015 of Finnish CMR practices provides valuable insights for their ongoing development. Attention should be paid to selection criteria for patients, patient involvement, and implementing and following up medication changes if recommended as a result of the medication review. Information from this study can be used beyond Finland for implementing new CMR practices in other countries. More studies need to be conducted on patient identification and follow-up to deepen understanding of these practices.

## Conclusions

Different types of CMR practices in varying health care settings were available and in routine use in Finland in 2015, the majority being designed for primary outpatient care and for comprehensive reviews of older patients’ medications. Even though practices might benefit from national standardization, allowing for flexibility in their customization according to context, medical and patient needs and available resources is important for optimizing care.

## Supplementary information

**Additional file 1.** Inventory Instrument.

## Data Availability

The data that support the findings of this study are available on request from the corresponding author AK. The data are not publicly available due to them containing information that could compromise research participant privacy.

## Abbreviations

AFP: The Association of Finnish Pharmacies
CMR: Collaborative Medication Review
EU: European Union
FIMEA: Finnish Medicines Agency
ILMA: The research project on medicines optimization for older adults
NHS: National Health Service
NICE: National Institute for Health and Care Excellence
PIM’s: Potentially inappropriate medications for older adults
SPSS-21: Statistical Package for the Social Sciences
UK: United Kingdom
USA: United States of America
