# Supplementary material for: An inventory of collaborative medication reviews for older adults - evolution of practices
Source: BMC Geriatr. 2019 Nov 21;19:321. doi: 10.1186/s12877-019-1317-6 (PMC6873748; doi:10.1186/s12877-019-1317-6)
Supplement: Supplementary file 1 — Additional file 1. Inventory Instrument. [file 12877_2019_1317_MOESM1_ESM.pdf]

INSTRUCTIONS FOR THE FILLING OF THE INVENTORY

The aim of this inventory is to identify collaborative medication review practices for optimization medications of the older adults. Own form should be filled from every separate practice / procedure / idea.

CONCEPTS

Collaborative practice / procedure refers to optimizing of the medications of the older adults in the multidisciplinary collaboration. Older adults refer to people aged 65-years or older.

Interprofessional collaboration refers to practice in which different healthcare professionals coordinate their actions to provide the best outcomes for the patients, understanding the tasks and responsibilities of other professionals in the treatment of the patient as well as the importance of information exchange between the professionals (Medicines Policy 2020 by the Ministry of Social Affairs and Health 2011).

Medication optimization means the patient care which acknowledges the patient individually and in which potential and/or actual medication-related problems are identified, the medication changes needed to solve the problems are made, and an attempt is made to prevent medication-related problems (Hepler and Strand 1990; Mäntylä 2012; Kallio 2014).

BACKGROUND INFORMATION

Organization____

Contact information____

(Company / Organization, Address, Postal code, Region, Respondent information: Phone number and E-mail)

FOLLOW-UP OF THE MEDICATIONS IN THE ORGANIZATION

1) How frequent are chronically ill patients' medications followed up with in your organization?
   a. Physician or nurse follow up visits once per year
   b. Prescription review is conducted at least once per year (concerns also patients whose medications have remained the same throughout the year)
   c. Follow-up of the high-alert medications (e.g., warfarin, NSAIDs)
   d. Follow-up is conducted elsewhere
   e. Do not know
   f. Other, please specify:____

2) Medication care plans are established conducted for:[1]
   a. All patients systematically
   b. Specific/selected patient groups systematically
   c. Some patients selectively
   d. Some patients randomly
   e. Are conducted elsewhere
   f. Are not conducted
   g. Other, please specify:____

3) To whom are medication lists reconciled in your organization: [1]
   a. To all patients systematically
   b. To specific/selected patient groups systematically
   c. To some patients selectively
   d. To some patients randomly
   e. Is reconciled elsewhere
   f. Other, please specify:____

DESCRIPTION OF THE COLLABORATIVE MEDICATION REVIEW PROCEDURE, PRACTICE OR IDEA.

4) Name of the procedure/practice/idea (if it has a specific name):
   ____

5) Is the collaborative medication review practice of your institution: [1]
   a. In the planning phase
   b. Under development
   c. In the pilot phase
   d. In routine use

6) The year of implementation: [1]
   a. 1999 or earlier
   b. 2000
   c. 2001
   d. 2002
   e. 2003
   f. 2004
   g. 2005
   h. 2006
   i. 2007
   j. 2008
   k. 2009
   l. 2010
   m. 2011
   n. 2012
   o. 2013
   p. 2014
   q. 2015

7) How widely is the collaborative medication review practice implemented?
   a. Only in our unit (all professionals involved are from the same unit)
   b. Only in our unit (based on collaboration between other organizations / units)
   c. The entire organization
   d. Throughout the federation of municipalities
   e. Throughout the hospital district
   f. Other, please specify:____

8) Context (choose all that apply): [1]
   a. University hospital
   b. Central hospital
   c. Other hospital
   d. Other inpatient care facility
   e. Assisted living at home
   f. Assisted living facilities
   g. Nursing home
   h. Primary outpatient care
   i. Community Pharmacy
   j. Other, please specify:____

9) Funding: [1]
   a. Government or municipality
   b. Private
   c. Public and private

10) Description of the medication review process: [1]
   - Where (e.g. at admission in the hospital), how (e.g. by using Medication Review checklist, trigger tools, Resident Assessment Instrument RAI) and by whom is a patient with medication-related problems identified?[2]
   - How do different health care professionals and organizations communicate patient information in different phases of the medication review process?[2]
   - What is the target group (e.g. patients with heart failure, aged customers in the pharmacy, discharged patients)?[2]
   - Where are the patient's medications reviewed (the site/venue and phase of the patient's care path)?[2]
   - How is the actual review of the medications performed (i.e., a brief narrative description of the actual practice)?[2]
   - What are the medication-related issues reviewed?[2]
   - Where are the medication changes documented (Patient information system)?[2]
   - How are medications reconciled?[2]
   - How are the results of the medication review communicated to the patient?[2]
   - How proxies are involved in the medication review?[2]
   - How is the follow-up of implementing medication changes organized (what is followed up, e.g. the effects of the medications changes, medication adherence; how and who is conducting the follow-ups)?[2]

11) Please define the purpose of the medication review practice (choose all that apply): [1]
   a. Technical review of prescriptions / list of medications (e.g. doses)
   b. Review of the prescriptions, the patient is involved to discuss medication taking and support adherence (e.g. taking medications as prescribed)
   c. Review of the medications in the context of the patient's clinical condition (e.g. access to laboratory test values/measures)

12) Which health care professionals are involved in reviewing the medications (choose all that apply):[1]
    a. Physician
    b. Nurse
    c. Practical nurse
    d. Pharmacist
    e. Physical therapist
    f. Other, please specify:____

13) How are the results of the medication reviews communicated and managed in the team (choose all that apply)? [1]
    a. Decisions were made in a team meeting
    b. Physician makes decisions individually for each case
    c. The results are not communicated for further actions Not dealt with
    d. Other, please specify:____

14) Medication review is conducted for:
    a. Specific patient groups systematically
    b. Some patients selectively
    c. Some patients randomly
    d. Other, please specify:____

15) Is the patient actively involved in the medication review process?[1]
    a. Yes
    b. No

16) How is the patient involved (choose all that apply)?[1]
    a. The patient is interviewed
    b. Home visit
    c. Medication care plan is discussed with the patient
    d. Patient keeps the medication list updated
    e. Other, please specify:____

17) What kind of tools are used in reviewing medications (electronic and/or manual) (choose all that apply)?: [1]
    a. Finnish Pharmaceutical Reference Book (Pharmaca Fennica®)
    b. Physician's manual
    c. Database to support physicians' clinical decision making (Physician's database®)
    d. Database to support nursing work (Nursing database®)
    e. Database for use of medicines in renal failure (Renbase®)
    f. Database for use of medicines during pregnancy and lactation (Gravbase and Lactbase®)
    g. Database on use of herbal medicines (Herbalbase®)
    h. Databases for interactions and cumulative adverse drug reactions (Sfinx-Pharao®)
    i. Database for interactions (Sfinx®)
    j. Medication review tool for community pharmacists (Salko®)

k. Database for identifying potentially inappropriate medicine use for the aged (Meds75+)
l. Medicines and laboratory results database
m. Other, Please specify:____

Evaluation of the Practice

18) How has this practice effected medication use in older adults? [2]

19) What are the strengths of your practice? [2]

20) What are the weaknesses or development needs of your practice? [2]

21) Has your practice been evaluated? If yes, how and how often? [2]

22) What are /were the challenges when implementing your practice? [2]

23) Is your practice based on any previous medication review procedure and/or theory? [2]

Future Plans

24) Describe your plans for further development of your practice in the future? [2]

25) Comments: [2]
____
____
____

[1]A question included in this study

[2]An open-ended question
